# Molecular pathways underlying inhibitory effect of antimicrobial peptide Nal-P-113 on bacteria biofilms formation of *Porphyromonas gingivalis* W83 by DNA microarray

**DOI:** 10.1186/s12866-017-0948-z

**Published:** 2017-02-17

**Authors:** Hong-yan Wang, Li Lin, Li-si Tan, Hui-Yuan Yu, Jya-Wei Cheng, Ya-ping Pan

**Affiliations:** 1 Department of Periodontics, School of Stomatology, China Medical University, Shenyang, 110002 China; 2 Institute of Biotechnology and Department of Medical Science, National Tsing Hua University, Hsinchu, 300 Taiwan

**Keywords:** *Porphyromonas gingivalis* W83, Nal-P-113, Microarray, Biofilms

## Abstract

**Background:**

Wound-related infection remains a major challenge for health professionals. One disadvantage in conventional antibiotics is their inability to penetrate biofilms, the main protective strategy for bacteria to evade irradiation. Previously, we have shown that synthetic antimicrobial peptides could inhibit bacterial biofilms formation.

**Results:**

In this study, we first delineated how Nal-P-113, a novel antimicrobial peptide, exerted its inhibitory effects on *Porphyromonas gingivalis* W83 biofilms formation at a low concentration. Secondly, we performed gene expression profiling and validated that Nal-P-113 at a low dose significantly down-regulated genes related to mobile and extrachromosomal element functions, transport and binding proteins in *Porphyromonas gingivalis* W83.

**Conclusions:**

These findings suggest that Nal-P-113 at low dose is sufficient to inhibit the formation of biofilms although *Porphyromonas gingivalis* W83 may maintain its survival in the oral cavity. The newly discovered molecular pathways may add the knowledge of developing a new strategy to target bacterial infections in combination with current first-line treatment in periodontitis.

**Electronic supplementary material:**

The online version of this article (doi:10.1186/s12866-017-0948-z) contains supplementary material, which is available to authorized users.

## Backgrounds

Periodontitis is one of the worldwide infectious diseases in humans, with approximately half of the adults experiencing some degrees of chronic periodontitis in developing countries, while 15% of United Kingdom population have developed severe periodontitis [1]. With an increase in aging population, the problem becomes more critical because elderly patients have compromised immune systems which predispose them to a higher risk of contracting bacterial infections. Reduced ability in tissue repairing further substantiates the problem. Antibiotics have been proven to work effectively against bacterial infections. However, the overuse of drugs clearly drives the evolution of bacteria resistance, endangering the efficacy of antibiotics. Therefore there is an emergent need to identify novel compounds to counteract bacterial infections. In general, conventional antibiotics are unable to penetrate biofilms. The formation of biofilms allows the bacteria to anchor and propagate in the tissue. Therefore, targeting the formation of biofilms may be a new therapeutic option for periodontitis.

Previous studies have shown that synthetic antimicrobial peptides inhibit bacterial biofilms formation. Numerous studies have confirmed that the major antimicrobial peptides mediated bactericidal mechanism is via rapid perforation of the cell membrane as well as activation of the apoptotic program by interrupting the normal physiological metabolism [2–4]. It has been demonstrated that antimicrobial peptide LL-37-treated *Pseudomonas aeruginosin* showed enormous changes in its gene transcription, with many de-regulated genes involved in the function of flagellar [5]. Similarly, antimicrobial peptide 1037 treatment for 24 h significantly changed the gene expression profiles knowing to be regulated by LL-37 treatment [6]. Nal-P-113, a modified version of antimicrobial peptide P-113, its amino sequence is AKR-Nal-Nal-GYKRKF-Nal-NH2. Antimicrobial peptide P-113 showed promising antimicrobial effects against a variety of pathogens [7–11]. Compared to P-113, Nal-P-113 maintained its effects when exposed to a high salt concentration and therefore it was an ideal candidate for application in complicated matrices including oral cavity, serum and plasma [12].

We have previously shown that Nal-P-113 exerts its anti-bactericidal effects in a rat periodontitis model with a significant reduction in tissue inflammation. Furthermore, we have found that Nal-P-113 inhibits *Porphyromonas gingivalis* (*P. gingivalis*) W83 biofilms formation. In this study, we aimed to determine the effects of different concentrations of Nal-P-113 in biofilms formation. Besides, we performed gene expression profiles in Nal-P-113-treated *P. gingivalis* W83 to delineate the underlying molecular mechanism of Nal-P-113-inhibited biofilms formation.

## Methods

### Bacteria strain

*P. gingivalis* W83 was a gift from Professor RJ Lamont (now in Department of oral Immunology and Infectious Disease, School of Dentistry, University of Louisville) from College of Dentist, University of Florida. Freshly prepared brain heart infusion (BHI, Difco Laboratories, MI, USA) agar medium supplemented with 5% sterile defibrinated sheep’s blood, 1% hemin, and 0.1% menadione, was used to grow *P. gingivalis* W83 at 37 °C under anaerobic conditions (80% N_2_, 10% H_2_ and 10% CO_2_) for 5 to 7 days.

### Reagents

Antimicrobial peptide Nal-P-113, Ac-AKR-Nal-Nal-GYKRKF-Nal-NH_2_, was provided by Prof. Jiawei Cheng in National Tsing Hua University [13]. H_2_O_2_ was purchased from Sigma Aldrich (CA).

### Bactericidal assay

*P. gingivalis* W83 was diluted to 5 × 10^5^ CFU/mL (CFU, colony forming units). The bacteria were treated with Nal-P-113 in 100 μL culture medium for 24 h. Then an aliquot (50 μL) of the resulting bacterial cell suspension was cultivated on a brain heart infusion agar plate. The bacterial cells were enumerated after incubation at 37 °C for 7 days. All experiments were repeated three times.

### Growth inhibition assay

*P. gingivalis* W83 culture was diluted to 5 × 10^5^ CFU/mL. The bacteria were treated with Nal-P-113 at different concentrations (0, 5, 10, 20, 40, 80, 160 and 320 μg/mL respectively) in 100 μL culture medium for 48 h. The cell growth was measured by the absorbance at 600 nm in a microplate reader (Tecan Infini M200, Switzerland). All experiments were repeated three times.

### Scanning electron microscopy (SEM) analysis on Biofilms

Biofilms formation was quantified on 6-well plates (Corning, Netherlands) which were coated with artificial saliva (Guangzhou Kodak Adhesives Co. Ltd., China). Five hundred microliter of *P. gingivalis* W83 (5 × 10^6^ CFU/mL) with or without 6.25 μg/mL Nal-P-113 treatment was dropped on 6-well plates and cultured for 48 h to establish biofilms. Then, the samples were fixed with 2.5% glutaraldehyde (BioChemika, Fluka, USA), washed with PBS and gradually dehydrated with ethanol. The processed samples were smeared onto copper plates followed by gold sputtering, and images were acquired using scanning electron microscopy (Inspect F50, FEI Company, USA) at 20,000 × magnification.

### Microarray hybridization

The total RNA of the bacteria treated with or without 6.25 μg/mL Nal-P-113 was extracted, converted to cDNA, labeled with Cy3-dCTP (GE, Healthcare, CA) and followed by hybridization with *P. gingivalis* W83 chip (Agilent, CA). Array hybridization, washing, scanning and data analysis were performed at the CapitalBio Corporation (Beijing, China).

### Data analysis

The array data were analyzed with the GeneSpring software V12 (Agilent). Significant genes were defined by following criterions: FDR < 5% and fold change > 2 [14, 15].

### Quantitative PCR (qPCR)

The total RNA of *P. gingivalis* W83 treated with or without 6.25 μg/mL Nal-P-113 was reversed transcribed into cDNA with the M-MLV RTase cDNA Synthesis Kit (Takara, China). Real-time quantitative PCR reaction was performed using SYBR Premix Ex TaqTM II PCR Master Mix Reagents Kit (Takara). The primers for the RT-qPCR were listed in Additional file 1: Table S1. qPCR was performed three times for each sample. Relative quantification of the mRNA levels was performed using the comparative Ct method with the formula 2^-ΔΔCt^.

### Hydrogen peroxide pre-treatment in *P. gingivalis*

*P. gingivalis* W83 was treated with H_2_O_2_ at different concentrations (0, 0.5, 1, and 3 mM, respectively) for 1 h. The H_2_O_2_ was washed away with phosphate buffer saline before subsequent experiments. Total RNA of the respective H_2_O_2_-treated *P. gingivalis* was extracted and followed by RT-qPCR to determine the mRNA expression of PG0841, PG0842, PG0872, PG0874, PG0875, PG1473, PG1474, PG1475, PG1478, PG1479, PG1482 and PG1485.

### Biofilms susceptibility assay

3 mM H_2_O_2_ was used to change transposase genes expression of *P. gingivalis* W83 for 1 h. The effect of Nal-P-113 on *P. gingivalis* W83 biofilms formation was examined using the microdilution method [12]. The resulting biofilms were fixed with 95% methanol and stained with 0.5% (w/v) crystal violet prior dissolving with 95% ethanol and subjected to microplate reader at absorption 590 nm. Percentage of inhibition was calculated using the equation [1-(A590 of the test/A590 of non-treated control)] × 100. All experiments were repeated three times.

### Enzyme linked immunosorbent assay (ELISA)

Lipopolysaccharide, free hemin and hemoglobin were measured by commercial ELISA kit according to the respective manual. Free hemin ELISA kit was purchase from Abnova, Taiwan, and the hemoglobin ELISA kit was purchased from Leagene, China. The measurements were analysis by Curve Expert 1.3 (AL).

### Statistical analysis

All experiments were performed in triplicate and repeated at least three times. Data were expressed as means ± standard deviations (SD). ANOVA and independent samples *t*-test were used to calculate the significance among the groups (SPSS Inc., IL, USA). *P*-value < 0.05 was considered statistically significant.

## Results

### The antibacterial activity of Nal-P-113 against *P. gingivalis* W83

**Fig. 1:**
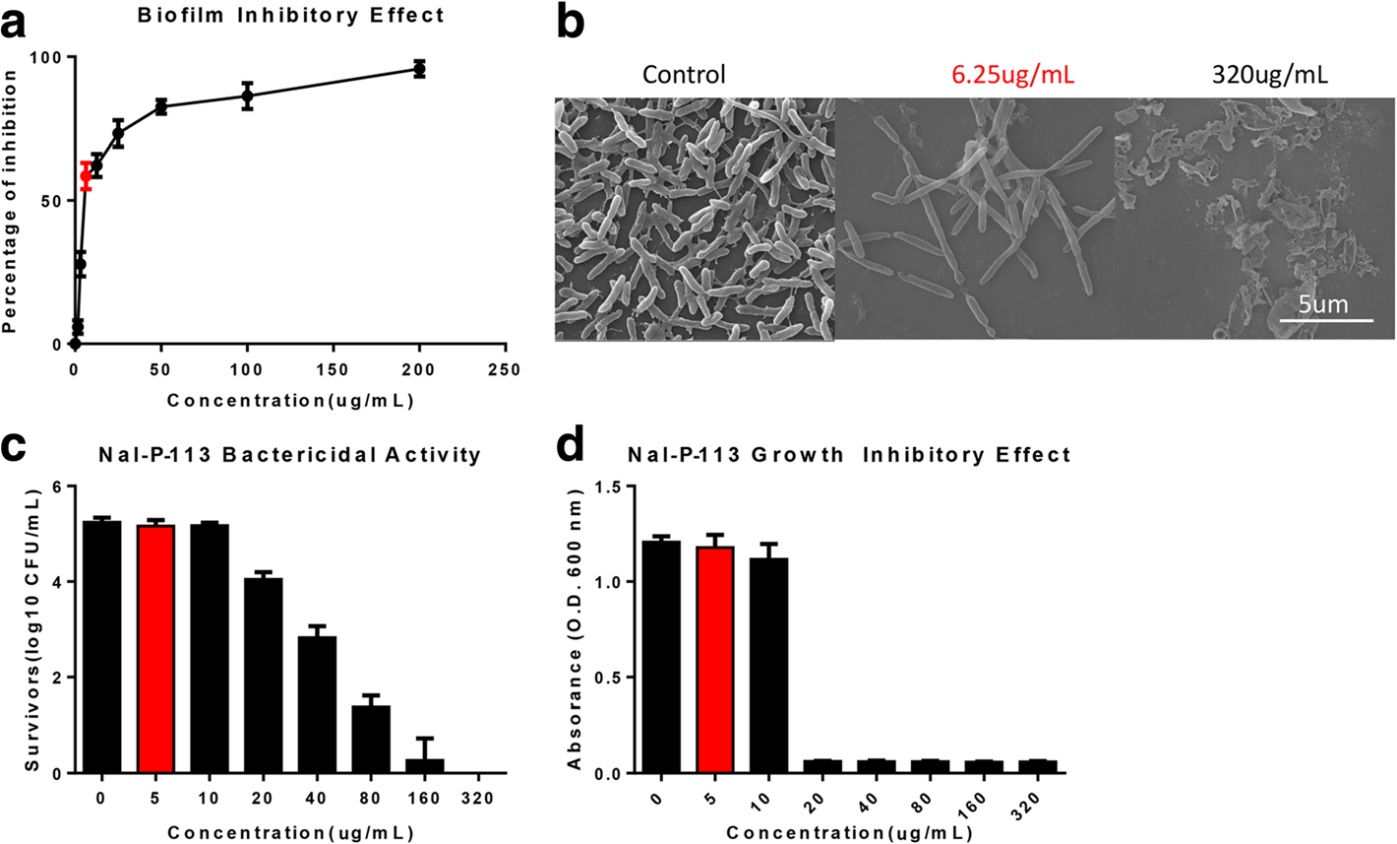
The MIC of Nal-P-113 to *P. gingivalis* W83 was 160 μg/mL, the MBC was 20 μg/mL, the MBIC was 6.25 μg/mL. The above results showed that Nal-P-113 inhibited *P. gingivalis* biofilms at a concentration that did not inhibit *P. gingivalis* growth. **a** Biofilms susceptibility assay of Nal-P-113 against *P. gingivalis* W83. **b** SEM image of *P. gingivalis* W83 in biofilm formation. **c** Bacterial growth assay of Nal-P-113-treated *P. gingivalis* W83. **d** Bactericidal assay of Nal-P-113-treated *P. gingivalis* W83. Data shown here are the mean ± S.D. from three independent cultures

*P. gingivalis* W83 is one of the major pathogenic species in dental plaque biofilms. Firstly, we examined the inhibitory effects of Nal-P-113 on *P. gingivalis* W83 biofilms formation. Consistent with our previous study, 6.25 μg/mL Nal-P-113 significantly inhibited *P. gingivalis* W83 biofilms formation (Fig. 1a). The biofilms inhibitory effect of Nal-P-113 was further validated with scanning electron microscopy (SEM) (Fig. 1b). Before treated with Nal-P-113, the bacteria engaged with each other and resulted in the formation of biofilms. Upon 6.25 μg/mL Nal-P-113 treatment, the bacteria continued to divide and were unable to fuse together, therefore inhibited the formation of biofilms. Conversely, treatment with 320 μg/mL Nal-P-113 resulted in a complete breakdown of the bacteria structure. Secondly, we evaluated the antibacterial activity of Nal-P-113 against planktonic *P. gingivalis* W83. Results showed that Nal-P-113 at a low concentration exhibited neither bactericidal activity nor growth inhibitory effect against *P. gingivalis* W83 (Fig. 1c, d). These results suggested that Nal-P-113 at high concentrations inhibited biofilms formation by direct killing of the bacteria, whereas Nal-P-113 at a low concentration adopted an alternative mechanism to inhibit the formation of biofilms in *P. gingivalis* W83.

### Nal-P-113 induced differential gene expression and disrupted the signaling pathways *in P. gingivalis* W83

**Fig. 2:**
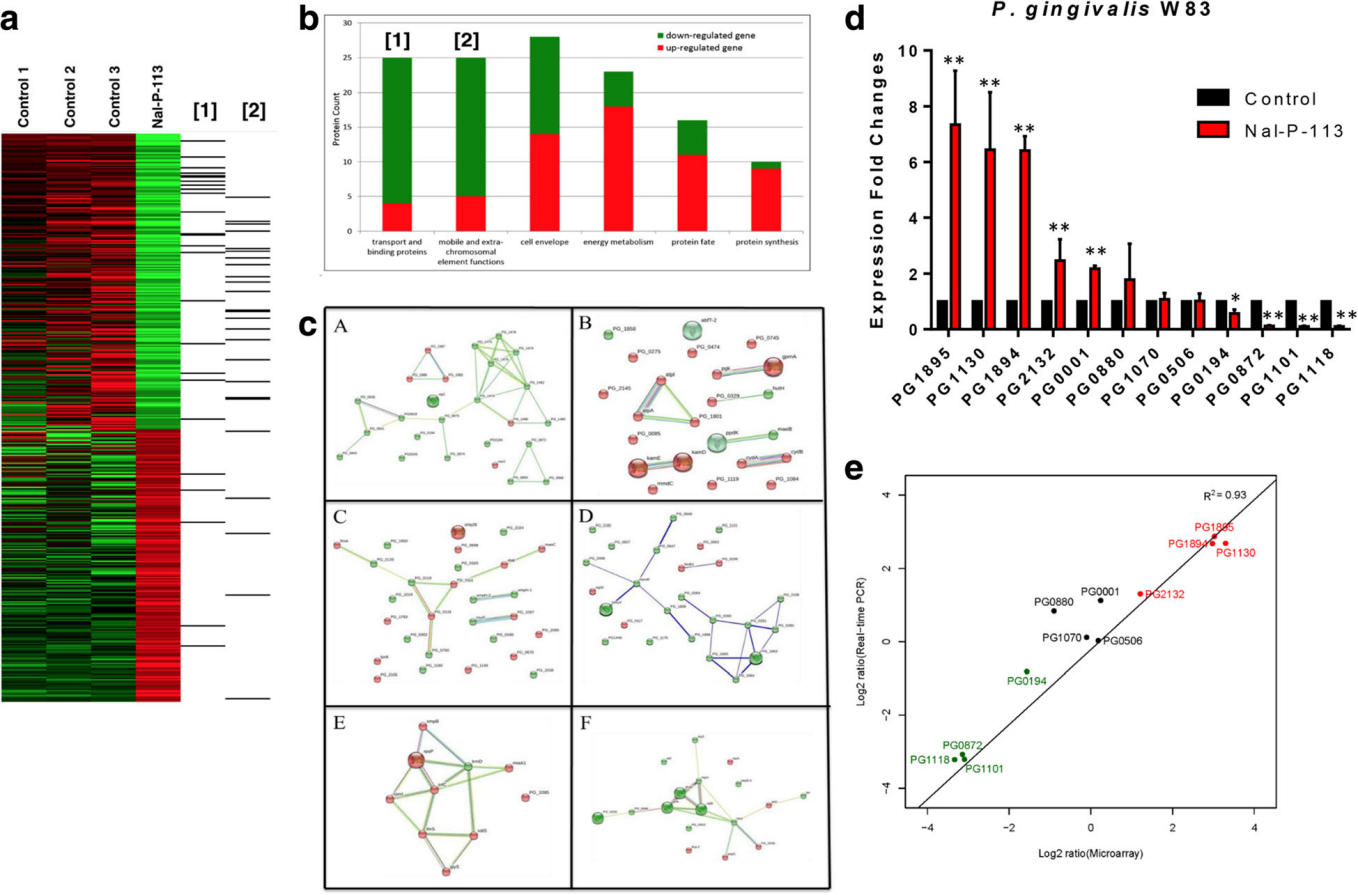
Differentially gene expression was exhibited in Nal-P-113-treated *Porphyromonas gingivalis* W83 as shown in KEGG pathway analysis. Differentially regulated genes can be categorized into six groups according to the TIGR genome database, including mobility and extrachromosomal element functions, energy metabolism, cell envelope, transport and binding proteins, protein synthesis and protein fate. 12 genes were selected to verify the consistency of quantitative PCR and Microarray analysis. **a** Supervised clustering of Nal-P-113-treated and non-treated *Porphyromonas gingivalis* (*n* = 3); (**b**) KEGG pathway analysis on the deferentially expressed genes in Nal-P-113-treated *Porphyromonas gingivalis*. **c** The network was constructed in the six major dys-regulated pathways. *a*: Differentially expressed genes related to mobile and extrachromosomal element functions. *b*: Differentially expressed genes related to energy metabolism. *c*: Differentially expressed genes related to cell envelope. *d*: Differentially expressed genes related to transport and binding protein. *e*: Differentially expressed genes related to protein synthesis. *f*: Differentially expressed genes related to protein fate. Nodes and edges represent differentially expressed genes and interactions among them. Up-regulated genes were represented as pink nodes, down-regulated genes were represented as green nodes. **d** The fold changes of gene expression levels in *Porphyromonas gingivalis* after Nal-P-113 treatment. Data shown here are the mean ± S.D. from three independent cultures. *, *P* < 0.05; **, *P* < 0.01. **e** Correlation of the expression fold change between quantitative PCR and microarray. Green, down-regulated genes, Black, unaffected genes, Red, up-regulated genes

To further understand the underlying mechanism of Nal-P-113-inhibited biofilms formation in *P. gingivalis* W83, we exposed the *P. gingivalis* W83 with 6.25 μg/mL Nal-P-113 for 0.5 h prior subjected to microarray gene analysis. Genes with expression fold change greater than 2 and FDR < 5% were identified as dysregulated genes. Supervised clustering analysis showed that Nal-P-113 treatment induced a significant alternation of gene expression in *P. gingivalis* W83 compared to untreated control (Fig. 2a). In total, Nal-P-113 treatment resulted in an increase in 204 transcripts and a decrease in 220 transcripts in *P. gingivalis* W83 (Additional file 1: Table S2). The complete list of gene expression values has been deposited in NCBI’s Gene Expression Omnibus (http://www.ncbi.nlm.nih.gov/geo/query/acc.cgi?acc=GSE 93873).

Although the genome of *P. gingivalis* W83 was published more than a decade ago, little is known about the interconnections among signaling pathways and how the interplay may impact the therapeutic interventions. We input the genes that were differentially expressed after Nal-P-113 treatment to STRING database for KEGG pathway analysis. Differentially regulated genes can be categorized into six groups according to the TIGR genome database. Majority of the dysregulated genes were involved in one of the following cellular functions/pathways: mobility and extrachromosomal element functions, energy metabolism, cell envelope, transport and binding proteins, protein synthesis and protein fate. Interestingly, dysregulated genes that were found to be involved in the down-regulated pathways were mainly related to mobility, extrachromosomal element functions as well as transport and binding protein. Meanwhile, differentially expressed genes involved in the up-regulated pathways were associated to cell envelope, energy metabolism, protein fate and synthesis (Fig. 2b and c).

Following, we selected the top 12 up-regulated and down-regulated genes (Additional file 1: Table S3) from the microarray analysis for validation. Apart from the dysregulated genes, we also included four additional genes whose expressions were not altered by Nal-P-113 as negative control. All top 6 up-regulated and down-regulated genes showed a similar trend and fold change in expression levels between microarray analysis and quantitative PCR, with a correlation value (R) of 0.964 (Fig. 2d and e).

### The biological effect of dysregulated genes and pathways after Nal-P-113 treatment

**Fig. 3:**
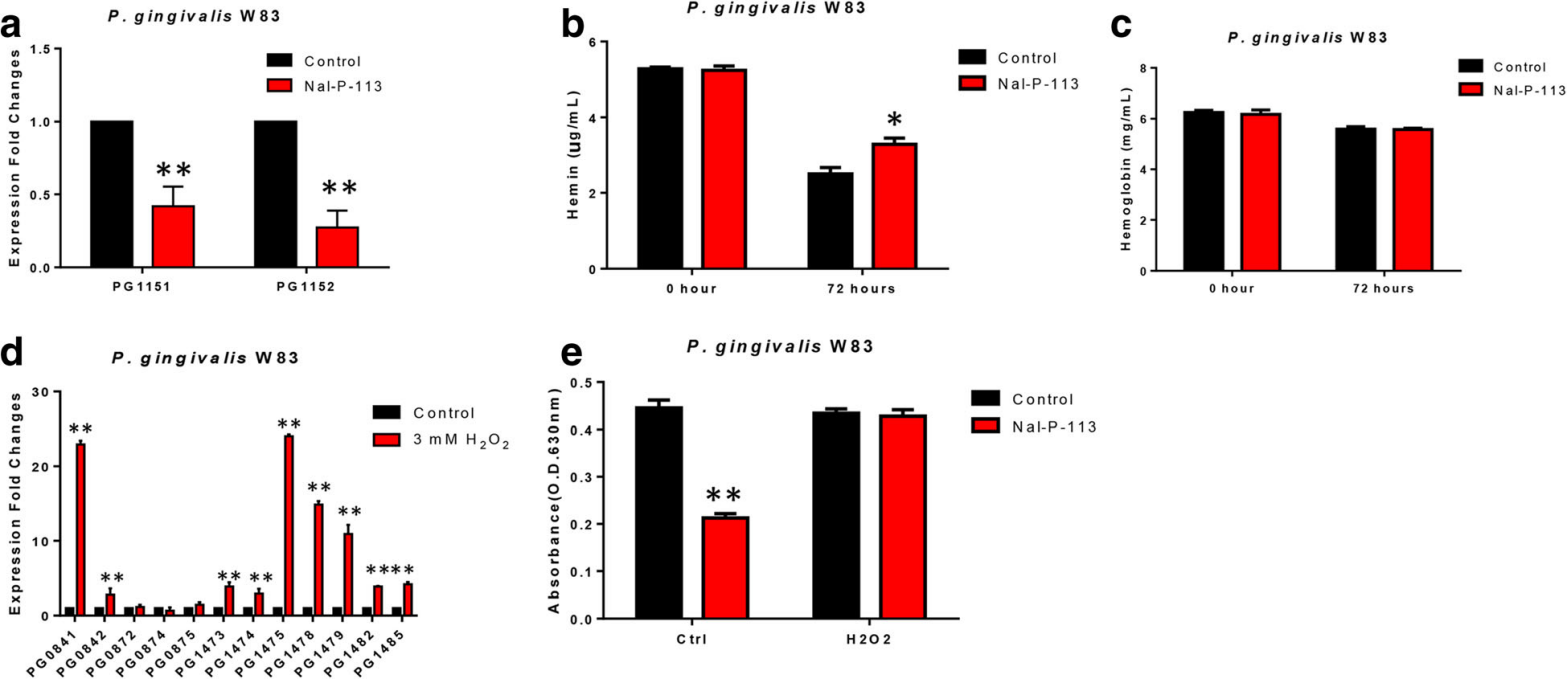
Validation of biological effect according to gene expression profiling analysis. **a** The fold changes of PG1151 and PG1152 gene expression levels in *Porphyromonas gingivalis* after Nal-P-113 treatment. **b** Free hemin concentration in *P. gingivalis* W83 culture medium either with or without treatment of Nal-P-113. **c** Hemoglobin concentration in *P. gingivalis* W83 culture medium either with or without treatment of Nal-P-113. **d** The expression of transposases levels in *Porphyromonas gingivalis* after 3 mM H_2_O_2_ treatment for one hour. **e** Biofilm susceptibility assay of Nal-P-113-treated *P. gingivalis* W83 with or without H_2_O_2_ pretreatment. Data shown here are the mean ± S.D. from three independent cultures, *, *P* < 0.05, **, *P* < 0.01

To further verify the underlying mechanism in Nal-P-113-treated *P. gingivalis* W83, we first used ELISA to detect free hemin and hemoglobin in liquid BHI. In accordance with the down-regulation of hmuY (PG1551) and HmuR (PG1552), known as proteins involved in the uptake of nutrients in a bacterial system (Fig. 3a), we observed a significant decrease in heme but not hemoglobin in the Nal-P-113-treated *P. gingivalis* W83 (Fig. 3b and c). Besides, when 3 mM H_2_O_2_ induced the expression of the transpoases genes that were found to be down-regulated by Nal-P-113 (Fig. 3d), 6.25 μg/mL Nal-P-113 did not inhibit *P. gingivalis* W83 biofilms formation (Fig. 3e). The result suggested that the transpoase pathways played a role in the Nal-P-113-regulated biofilms formation in *P. gingivalis* W83.

## Discussion

*P. gingivalis* is a Gram-negative oral anaerobe that has been known to play a major role in the pathogenesis of periodontitis. *P. gingivalis*, presents within the forming biofilms, orchestrates the virulence of the biofilms and consequent tissue inflammatory response to destroy the gingival tissues and ultimately resulting in tooth loss. Previously, we and others have shown that synthetic antimicrobial peptides can inhibit bacterial biofilms formation [12]. Multiple lines of evidence suggested that antimicrobial peptides acted via rapid perforation of the cell membrane [2–4]. Nevertheless, our SEM image showed that the cell membrane structure remained intact after treatment of 6.25 μg/mL Nal-P-113, indicating that a low concentration of Nal-P-113 did not disrupt the bacteria membrane. Nevertheless, Nal-P-113 at a low dose may still have indirect effects on bacterial membrane. For example, PG0192 and PG0193, encoding the cationic outer membrane proteins that involve in the colonization and biofilms formation, were both down-regulated by 2.15 fold and 2.07 fold, respectively, upon Nal-P-113 treatment. In parallel, PG2133 encoding lipoprotein was down-regulated by 2.50 fold. The Gram-negative bacterial lipoprotein is an essential component of the outer membrane, which plays a key role in bacterial infection, survival, as well as invading and damaging the host’s immune defense. The down-regulation of PG0192, PG0193 and PG2133 may be correlated with the effects of antimicrobial peptides in the bacterial membranes that reduce their ability to maintain cellular structure, to form biofilms or to survive.

Studies have reported the inhibiting effects of antimicrobial peptides in biofilms formation, which involved the disruption of the bacteria’s physiological metabolism [2–4]. However, our gene expression profiling demonstrated that the presence of Nal-P-113 enhanced *P. gingivalis* W83 aerobic respiration to survive. There were 23 differentially expressed genes function in energy metabolism, five genes were down-regulated, and 18 genes were up-regulated. Cytochrome dubiquinol oxidase-encoding genes, cydA (PG0900) and cydB (PG0899), which are related to aerobic respiration in *P. gingivalis* [16], were up-regulated by 3.64-fold and 2.06–fold, respectively. Within the locus encoding cydA and cydB, there is an open reading frame encoding a protein of unknown function (PG0901), and was up-regulated in the presence of Nal-P-113, indicating that it may be a member of the same transcriptional unit as the cydAB operon. PG0130, encoding the phosphoglycerate mutase that participates in DNA and RNA synthesis, was up-regulated. Furthermore, genes related to protein synthesis were up-regulated. PG0502, encoding SsrA-binding protein, was up-regulated by 2.61-fold. PG0992, encodes threonyl-tRNA synthetase, PG1132 encodes valyl-tRNA synthetase, and PG2165 encodes glycyl-tRNA synthetase, were found to be up-regulated. Ammonia acyl-tRNA synthetase is one of the key enzymes in protein synthesis [17]. PG2117 and PG0990 both encoding ribosomal proteins were shown to be up-regulated by 3.32-fold and 2.52-fold, respectively.

Moreover, transporting and binding protein pathway was also significantly inhibited by Nal-P-113 treatment. 4 out of the 25 transporting and binding protein-related genes were up-regulated, and the remaining 19 genes were down-regulated. PG0282 and PG1663 encode ABC transporter and ATP-binding protein, respectively. PG0281, PG0280, PG1664 and PG1665, encoding ABC transporter and permease proteins, were down-regulated. ABC transporters are a group of transmembrane proteins, known to be associated with a variety of physiological processes including the uptake of nutrients, the non-classical secretion of signaling molecules and toxins, multidrug resistance and the development of human disease. ABC transporters play a role in cell-to-surface and cell-to-cell interactions in biofilms development [18–20]. The observed differentially expressed genes in ABC transporter superfamily were in line with our SEM result, demonstrating the loss of cell contact in *P. gingivalis* W83 after treated with Nal-P-113 at a low concentration.

PG2016, encoding CRISPR-associated helicase, PG1985, PG1986 and PG1987 encoding other CRISPR-associated proteins, were shown to be up-regulated and may be related to the immune response due to foreign DNA. The down-regulation in integrase and the up-regulation of CRISPR-associated proteins indicated that the killing of *P. gingivalis* W83 by Nal-P-113 differed to that of antibiotics. Therefore, the acquisition of resistance to Nal-P-113 is less likely compared to antibiotic since different mechanisms are activated in the bacteria system.

Lastly, PG1551 encodes hmuY protein, while PG1552 encodes the TonB-dependent receptor HmuR. These two genes were down-regulated by 2.39-fold and 3.67-fold, respectively. The bacterial uptake system for heme requires two kinds of proteins: HmuY, which scavenges heme from host hemoproteins, and HmuR, which transports nutrients across the bacterial cell membrane. The down-regulation of these two genes by Nal-P-113 suggested that Nal-P-113 treatment might weaken the ability of *P. gingivalis* W83 to utilize hemin. In fact, our ELISA assays verified that the concentration of hemin decreased significantly in the Nal-P-113-treated *P. gingivalis* W83 liquid BHI, indicating that Nal-P-113-treated bacteria could not utilize the hemin efficiently, therefore inhibited the formation of biofilms. Furthermore, PG0841, PG0842, PG0872, PG0874, PG0875, PG1473, PG1474, PG1475, PG1478, PG1479, PG1482, PG1485, which encode the transposase genes were all down-regulated upon Nal-P-113 treatment. Previous studies have shown that H_2_O_2_ induces the expression of transposase genes in *P. gingivalis* [21, 22]. Our results showed that H_2_O_2_ pre-treatment abolished 6.25 mg/mL Nal-P-113 anti-biofilms formation in *P. gingivalis* W83, which indicated that transposase genes played an important role in Nal-P-113-mediated biofilms formation.

## Conclusions

*P. gingivalis* is one of the major etiologic agents which contributes to chronic periodontitis. It attributes to the formation of subgingival biofilms and stimulates a cascade of inflammatory reaction. Our results showed that Nal-P-113 exerted its anti-microbial and anti-biofilms effects in *P. gingivalis* W83 by mediating the energy metabolism, protein synthesis, mobile and extrachromosomal element functions, transport and binding proteins of the bacteria. In addition, we verified that Nal-P-113 acted through the down-regulation of transposase genes or reduction of hemin utilization in *P. gingivalis* W83 to inhibit the formation of biofilms. In summary, this study provides a molecular biological basis of Nal-P-113 in the prevention and treatment of periodontitis.

## Additional file

Additional file 1: Table S1. List of primers used for qPCR experiments. **Table S2**. Up-regulated gene list in Nal-P-113 treated *P.gingivalis* W83 (hypothetical protein excluded). **Table S3**. Down-regulated gene list in Nal-P-113 treated *P.gingivalis* W83 (hypothetical protein excluded). (DOCX 46 kb)

## Abbreviations

BHI: Brain heart infusion
CFU: Colony forming units
CRISPR: Clustered regularly interspaced short palindromic repeat
ELISA: Enzyme linked immunosorbent assay
LPS: Lipopolysaccharide
qPCR: Quantitative PCR
SEM: Scanning electron microscopy
