# Supplementary material for: Molecular pathways underlying inhibitory effect of antimicrobial peptide Nal-P-113 on bacteria biofilms formation of Porphyromonas gingivalis W83 by DNA microarray
Source: BMC Microbiol. 2017 Feb 17;17:37. doi: 10.1186/s12866-017-0948-z (PMC5316201; doi:10.1186/s12866-017-0948-z)
Supplement: Additional file 1: Table S1. — List of primers used for qPCR experiments. Table S2. Up-regulated gene list in Nal-P-113 treated P.gingivalis W83 (hypothetical protein excluded). Table S3. Down-regulated gene list in Nal-P-113 treated P.gingivalis W83 (hypothetical protein excluded). (DOCX 46 kb) [file 12866_2017_948_MOESM1_ESM.docx]

**Supplementary Table 1***.* List of selected genes and primers for qPCR

| Gene |  | Primers | Amplicon (bp) |
| --- | --- | --- | --- |
| PG1130 | -F | 5’-CAAAAGCGGAACAGGCCATC-3’ | 72 |
|  | -R | 5’-GCAATATCTCGCGCAAAGGG-3’ |  |
| PG1895 | -F | 5’- TGACAGTGGATCTGGCACAC-3’ | 87 |
|  | -R | 5’- CAGGGCGCAAATAAGGCATC-3’ |  |
| PG1894 | -F | 5’-CCTATCCTACTGTTTCAAGGCCA-3’ | 77 |
|  | -R | 5’-TTTGGGCTGTTTCCGCATAA-3’ |  |
| PG2132 | -F | 5’- CAAAACGGGGATTTCATTTCTC-3’ | 147 |
|  | -R | 5’- GCCTATGACGGGTATGAATGGT-3’ |  |
| PG0506 | -F | 5’- AACCAGTCTTGGGCTTCTCCT-3’ | 82 |
|  | -R | 5’- GCTTGATGTTGTTCGGGTGTT-3’ |  |
| PG0872 | -F | 5’-CGCTCCACGATTCAGTGCTA-3’ | 74 |
|  | -R | 5’-CACAGCTTATCACGCTGGGA-3’ |  |
| PG1118 | -F | 5’- TCATCGAAAAGCACCACGGA-3’ | 81 |
|  | -R | 5’- GGGGTATGTGGGCTATGACG-3’ |  |
| PG1101 | -F | 5’-TCTGATTGCTCAAGCGCAGA-3’ | 99 |
|  | -R | 5’-ATACGGACTGGCGCATACAG-3’ |  |
| PG0194 | -F | 5’-CGCAGAATCTGACCGAAATA-3’ | 146 |
|  | -R | 5’-CATCTACGATGCCAAGCGA-3’ |  |
| PG0880 | -F | 5’-GGCTACGAAATCATCGGAGT-3’ | 110 |
|  | -R | 5’-TTGAGACGCACTTCCTCATC-3’ |  |
| PG1070 | -F | 5’-TTACACTCACCGCTGAAGAAGAAG-3’ | 100 |
|  | -R | 5’-TTCTCGGGGTCTATCAAACTCAA-3’ |  |
| PG0001 | -F | 5’-AGGTGGTCATGTTCCTCTCC-3’ | 78 |
|  | -R | 5’-GGTTACGTCCTCCCATCAGT-3’ |  |
| PG0841 | -F | 5’-GGTATTGCTCCCCTCTACGC-3’ | 124 |
|  | -R | 5’-TGGAGAGTGCAAACGAGGTC-3’ |  |
| PG0842 | -F | 5’-AGAGCCATCATCCGCTATGC-3’ | 87 |
|  | -R | 5’- GATGTCGTGCATCAAGTCGC-3’ |  |
| PG0872 | -F | 5’- CGAAAGGGTGCAACTACAGC-3’ | 71 |
|  | -R | 5’- TTACGACGCACCTCTTGACG-3’ |  |
| PG0874 | -F | 5’- CCTTCTCAACGCCAAGCAAC-3’ | 113 |
|  | -R | 5’- GCCACTTATCTCGGTACGCA-3’ |  |
| PG0875 | -F | 5’- ACTGTTCGGGAGGCTATGGA-3’ | 106 |
|  | -R | 5’- TGAGGACATTCCGACCTTGC-3’ |  |
| PG1473 | -F | 5’- AAGGTGTTTTGCGGATCGGA-3’ | 112 |
|  | -R | 5’- GACTGCCGCTCTTTGCTTTG-3’ |  |
| PG1474 | -F | 5’- TCTTGGATCGTTCGGGCTTC-3’ | 87 |
|  | -R | 5’- TGACGAGCAGCAAGAGACTG-3’ |  |
| PG1475 | -F | 5’- CAACGGGCTGCTTTTCTTCC-3’ | 72 |
|  | -R | 5’- GTGATGAAGTCCACCGAGCA-3’ |  |
| PG1478 | -F | 5’- TCGCTTATGCTCGCTCTCAG-3’ | 103 |
|  | -R | 5’- CCGGCGCAATCGTAAAGAAG-3’ |  |
| PG1479 | -F | 5’- GGAAGCCTGTAAACGGGACA-3’ | 96 |
|  | -R | 5’- TCTTCCGGGCCGATAAAACC-3’ |  |
| PG1482 | -F | 5’- ATGTGCGGCATGAACCAGTA-3’ | 113 |
|  | -R | 5’- TTCATCAGCCCGTGTTCTCC-3’ |  |
| PG1485 | -F | 5’- TGAAGAACAGCGCCAAAAGC-3’ | 148 |
|  | -R | 5’- CATCGTGATCTCCTGACGGG-3’ |  |

**Supplementary Table 2**. Up-regulated gene list in Nal-P-113 treated *P.gingivalis* W83 (hypothetical protein excluded)

| Locus no.^a^ | Putative identification^a^ | Cellular role^a^ | Fold^b^ |
| --- | --- | --- | --- |
| PG1130 | TPR domain-containing protein | Unknown function | 9.8027 |
| PG1469 | TypeⅠrestriction-modification system | DNA metabolism | 6.3215 |
| PG1501 | TetR family transcriptional regulator | Regulatory functions | 5.3752 |
| PG1428 | RibE | Biosynthesis of cofactors, prosthetic groups, and carriers | 5.3282 |
| PG0512 | Guanylate kinase | Purines, pyrimidines, nucleosides, and nucleotides | 4.3412 |
| PG2105 | Putative lipoprotein | Cell envelope | 4.2160 |
| PG1279 | D-isomer specific 2-hydroxyacid dehydrogenase family protein | Unknown function  Unknown function | 4.1214 |
| PG1864 | Leucine-rich protein | Unknown function | 3.9009 |
| PG0111 | Capsular polysacharride biosynthesis gene, putative | Cell envelope | 3.8617 |
| PG0209 | Formate/nitrite transporter | Transport and binding proteins | 3.8084 |
| PG2095 | Putative lipoprotein | Cell envelope | 3.7165 |
| PG0900 | cytochrome d ubiquinol oxidase, subunit I | Energy metabolism | 3.6436 |
| PG0086 | ATP-dependent RNA helicase, DEAD/DEAH box family | Transcription | 3.5937 |
| PG0698 | Putative lipoprotein | Cell envelope | 3.4023 |
| PG0991 | translation initiation factor IF-3 | Protein synthesis | 3.3880 |
| PG2117 | ribosomal protein S16 | Protein synthesis | 3.3163 |
| PG1203 | transcriptional regulator, putative | Regulatory functions | 3.3150 |
| PG0156 | modification methylase, HemK family | Unknown function | 3.3069 |
| PG1075 | coenzyme A transferase, beta subunit | Central intermediary metabolism | 3.3031 |
| PG1504 | NAD dependent protein | Unknown function | 3.2767 |
| PG0862 | type IIS restriction endonuclease, putative | DNA metabolism | 3.2367 |
| PG1358 | acetyltransferase, GNAT family | Unknown function | 3.2165 |
| PG1505 | radical SAM domain protein | Unknown function | 3.1942 |
| PG2016 | CRISPR-associated helicase Cas3 | Mobile and extrachromosomal element functions | 3.1312 |
| PG0992 | threonyl-tRNA synthetase | Protein synthesis | 3.1183 |
| PG1801 | v-type ATPase, subunit E, putative | Energy metabolism | 2.9986 |
| PG2162 | lipid A disaccharide synthase | Cell envelope | 2.9649 |
| PG0330 | DNA-binding protein, histone-like family | DNA metabolism | 2.9569 |
| PG0627 | RNA-binding protein | Transcription | 2.9548 |
| PG1258 | DNA-binding protein HU | DNA metabolism | 2.9530 |
| PG1095 | RNA methyltransferase, TrmA family | Protein synthesis | 2.9142 |
| PG1254 | acetyltransferase, GNAT family | Unknown function | 2.9133 |
| PG1509 | HAD-superfamily hydrolase, subfamily IA, variant1 family protein | Unknown function | 2.8923 |
| PG0324 | histidine ammonia-lyase | Energy metabolism | 2.8473 |
| PG0475 | oxygen-independent coproporphyrinogen III oxidase, putative | Biosynthesis of cofactors, prosthetic groups, and carriers | 2.8305 |
| PG0110 | glycosyl transferase, group 1 family protein | Cell envelope | 2.8123 |
| PG0329 | formiminotransferase-cyclodeaminase-related  protein" | Energy metabolism | 2.7855 |
| PG1598 | lipoprotein signal peptidase, putative | Protein fate | 2.7248 |
| PG1560 | dTDP-glucose 4,6-dehydratase | Cell envelope | 2.7059 |
| PG0543 | transcriptional regulator, putative | Regulatory functions | 2.6672 |
| PG0118 | glycosyl transferase, group 2 family protein | Cell envelope | 2.6463 |
| PG1255 | recombination protein RecR | DNA metabolism | 2.6348 |
| PG1149 | "glycosyl transferase, group 1 family protein | Cell envelope | 2.6194 |
| PG0085 | alpha-galactosidase | Energy metabolism | 2.6136 |
| PG1542 | collagenase | Protein fate | 2.6117 |
| PG2145 | polysaccharide deacetylase | Energy metabolism | 2.6109 |
| PG0502 | SsrA-binding protein | Protein synthesis | 2.6051 |
| PG0087 | SIS domain protein | Unknown function | 2.5888 |
| PG2161 | transcriptional regulator, AraC family | Regulatory functions | 2.5857 |
| PG1097 | Mur ligase domain protein/alanine racemase | Cell envelope | 2.5851 |
| PG0615 | GTP-binding protein TypA | Unknown function | 2.5622 |
| PG0990 | ribosomal protein L35 | Protein synthesis | 2.5188 |
| PG2173 | outer membrane lipoprotein Omp28 | Cell envelope | 2.5152 |
| PG1074 | D-lysine 5,6-aminomutase, beta subunit | Energy metabolism | 2.5062 |
| PG0754 | DNA topoisomerase I | DNA metabolism | 2.5061 |
| PG1503 | LytB-related protein | Unknown function | 2.5051 |
| PG1213 | ribonuclease H | Transcription | 2.4960 |
| PG0275 | thioredoxin family protein | Energy metabolism | 2.4940 |
| PG1985 | CRISPR-associated protein, TM1792 family | Mobile and extrachromosomal element functions | 2.4517 |
| PG0745 | lactoylglutathione lyase, putative | Energy metabolism | 2.4516 |
| PG1609 | methylmalonyl-CoA decarboxylase, gamma subunit | Energy metabolism | 2.4382 |
| PG1677 | phosphoglycerate kinase | Energy metabolism | 2.4363 |
| PG1605 | aminopeptidase C | Protein fate | 2.4299 |
| PG1544 | yaaA protein | Unknown function | 2.4268 |
| PG0130 | phosphoglycerate mutase | Energy metabolism | 2.3968 |
| PG1987 | CRISPR-associated protein, TM1811 family | Mobile and extrachromosomal element functions | 2.3919 |
| PG1886 | GTP-binding protein HflX | Unknown function | 2.3917 |
| PG1404 | rhomboid family protein | Unknown function | 2.3833 |
| PG1803 | v-type ATPase, subunit A | Energy metabolism | 2.3762 |
| PG1084 | thioredoxin family protein | Energy metabolism | 2.3623 |
| PG0474 | low-specificity L-threonine aldolase | Energy metabolism | 2.3514 |
| PG0885 | phospho-2-dehydro-3-deoxyheptonate aldolase/chorismate mutase | Amino acid biosynthesis | 2.3311 |
| PG1294 | ferrous iron transport protein B | Transport and binding proteins | 2.3219 |
| PG1072 | MutS family protein | DNA metabolism | 2.3139 |
| PG2132 | fimbrilin | Cell envelope | 2.3087 |
| PG2179 | NADH:ubiquinone oxidoreductase, Na  translocating, D subunit | Transport and binding proteins | 2.2956 |
| PG2072 | UvrD/REP helicase domain protein | Unknown function | 2.2823 |
| PG1135 | bacterial sugar transferase | Cell envelope | 2.2802 |
| PG1403 | rhomboid family protein | Unknown function | 2.2789 |
| PG1783 | glycosyl transferase, group 2 family protein | Cell envelope | 2.2584 |
| PG1806 | glycosyl transferase, group 2 family protein | Energy metabolism | 2.2579 |
| PG1728 | cytidine/deoxycytidylate deaminase family  protein | Unknown function | 2.2574 |
| PG0235 | carboxyl-terminal protease | Protein fate | 2.2512 |
| PG0670 | lipoprotein, putative | Cell envelope | 2.2089 |
| PG1119 | flavodoxin, putative | Energy metabolism | 2.2070 |
| PG1727 | yitL protein | Unknown function | 2.1847 |
| PG1073 | D-lysine 5,6-aminomutase, alpha subunit | Energy metabolism | 2.1797 |
| PG1789 | peptidyl-dipeptidase Dcp | Protein fate | 2.1598 |
| PG0456 | PHP N-terminal domain protein | Unknown function | 2.1534 |
| PG1114 | aspartate-1-decarboxylase | Biosynthesis of cofactors, prosthetic groups, and carriers | 2.1344 |
| PG1402 | AP endonuclease domain protein | Unknown function | 2.1202 |
| PG1145 | long-chain-fatty-acid--CoA ligase, putative | Fatty acid and phospholipid metabolism | 2.1190 |
| PG1854 | 5-formyltetrahydrofolate cyclo-ligase family  protein | Biosynthesis of cofactors, prosthetic groups, and carriers | 2.1124 |
| PG1486 | conjugative transposon protein TraA | Mobile and extrachromosomal element functions | 2.1122 |
| PG0850 | DNA binding protein, excisionase family,  putative | Unknown function | 2.1051 |
| PG1986 | DNA binding protein, excisionase family,  putative | Mobile and extrachromosomal element functions | 2.1051 |
| PG2165 | glycyl-tRNA synthetase | Protein synthesis | 2.0953 |
| PG1697 | type II restriction endonuclease, putative | DNA metabolism | 2.0880 |
| PG0959 | ATP-binding protein, Mrp/Nbp35 family | Unknown function | 2.0850 |
| PG0630 | pyridoxal phosphate biosynthetic protein PdxJ | Biosynthesis of cofactors, prosthetic groups, and carriers | 2.0837 |
| PG1640 | DNA-damage-inducible protein F | DNA metabolism | 2.0810 |
| PG0971 | McrBC restriction endonuclease system, McrB subunit, putative | DNA metabolism | 2.0724 |
| PG0335 | tRNA delta(2)-isopentenylpyrophosphate  transferase | Protein synthesis | 2.0700 |
| PG0508 | HAD-superfamily subfamily IB hydrolase,  TIGR01490 | Unknown function | 2.0679 |
| PG2163 | stationary-phase survival protein SurE | Cellular processes | 2.063 |
| PG0899 | cytochrome d ubiquinol oxidase, subunit II | Energy metabolism | 2.0608 |
| PG1076 | acyl-CoA dehydrogenase, short-chain specific | Fatty acid and phospholipid metabolism | 2.0411 |
| PG1856 | cytidine/deoxycytidylate deaminase family  protein | Unknown function | 2.0250 |
| PG0063 | outer membrane efflux protein | Transport and binding proteins | 2.0192 |
| PG0477 | pantoate--beta-alanine ligase | Biosynthesis of cofactors, prosthetic groups, and carriers | 2.0160 |
| PG0117 | "polysaccharide transport protein, putative | Transport and binding proteins | 2.0144 |
| PG1430 | TPR domain protein | Unknown function | 2.0141 |
| PG1604 | aminopeptidase C | Unknown function | 2.0109 |
| PG0433 | tetrapyrrole methylase family protein | Unknown function | 2.0095 |
| PG1190 | glycerate dehydrogenase | Central intermediary metabolism | 2.0057 |
| PG1132 | valyl-tRNA synthetase | Protein synthesis | 2.0022 |

^a^: Locus number, putative identification and cellular role are according to TIGR genome database.

^b^: Average fold difference indicates the expression of the gene by antimicrobial peptide Nal-P-113 addition versus no antimicrobial peptide Nal-P-113 addition.

**Supplementary Table 2.** Down-regulated gene list in Nal-P-113 treated *P.gingivalis* W83 (hypothetical protein excluded)

| Locus no.^a^ | Putative identification^a^ | Cellular role^a^ | Fold^b^ |
| --- | --- | --- | --- |
| PG1118 | clpB protein | Protein fate | -10.0775 |
| PG0872 | mobilizable transposon, xis protein | Mobile and extrachromosomal element functions | -8.8257 |
| PG1101 | sodium:solute symporter family protein | Transport and binding proteins | -8.5259 |
| PG0842 | mobilizable transposon, hypothetical protein,  putative | Mobile and extrachromosomal element functions | -7.6957 |
| PG0875 | mobilizable transposon, tnpA protein | Mobile and extrachromosomal element functions | -7.6514 |
| PG1495 | DNA topoisomerase III | DNA metabolism | -6.2556 |
| PG0174 | pyridine nucleotide-disulphide oxidoreductase family protein | Unknown function | -.5.6686 |
| PG1482 | conjugative transposon protein TraF | Mobile and extrachromosomal element functions | -.5.4728 |
| PG1475 | conjugative transposon protein TraN | Mobile and extrachromosomal element functions | -.5.1185 |
| PG0985 | RNA polymerase sigma-70 factor, ECF subfamily | Transcription | -4.8598 |
| PG1314 | chorismate synthase | Amino acid biosynthesis | -.4.4997 |
| PG0874 | mobilizable transposon, int protein | Mobile and extrachromosomal element functions | -4.4479 |
| PG0868 | mobilization protein | Mobile and extrachromosomal element functions | -.4.4268 |
| PG1055 | thiol protease | Protein fate | -.4.0681 |
| PG1446 | MATE efflux family protein | Transport and binding proteins | -4.0615 |
| PG0841 | mobilizable transposon, excision protein,  putative | Mobile and extrachromosomal element functions | -4.0518 |
| PG0869 | mobilization protein | Mobile and extrachromosomal element functions | -4.0194 |
| PG0214 | RNA polymerase sigma-70 factor, ECF subfamily | Transcription | -3.8637 |
| PG0593 | htrA protein | Protein fate | -3.7909 |
| PG1479 | conjugative transposon protein TraJ | Mobile and extrachromosomal element functions | -3.7460 |
| PG2028 | ebsC protein | Unknown function | -3.7243 |
| PG1180 | membrane protein, putative | Cell envelope | -.3.6958 |
| PG1552 | TonB-dependent receptor HmuR | Transport and binding proteins | -3.6750 |
| PG0180 | lipoprotein, putative | Cell envelope | -3.6339 |
| PG0183 | lipoprotein, putative | Cell envelope | -.3.6269 |
| PG0537 | aminoacyl-histidine dipeptidase | Protein fate | -3.6177 |
| PG1579 | ATPase, MoxR family | Unknown function | -.3.5963 |
| PG0819 | integrase | Mobile and extrachromosomal element functions | -.3.5817 |
| PG2109 | hydroxymethylpyrimidine kinase/thiamin-phosphate pyrophosphorylase | Biosynthesis of cofactors, prosthetic groups, and carriers | -.3.5777 |
| PG1858 | flavodoxin | Energy metabolism | -3.5697 |
| PG0826 | transcriptional regulator, AraC family | Regulatory functions | -.3.5625 |
| PG2008 | TonB-dependent receptor, putative | Transport and binding proteins | -.3.5175 |
| PG0222 | DNA-binding protein, histone-like family | DNA metabolism | -.3.5028 |
| PG0827 | MATE efflux family protein | Transport and binding proteins | -.3.4251 |
| PG1099 | glucokinase regulator-related protein | Unknown function | -.3.4184 |
| PG1105 | RNA polymerase sigma-54 factor | Transcription | -.3.3955 |
| PG2201 | polypeptide deformylase | Protein fate | -.3.3922 |
| PG1208 | dnaK protein | Protein fate | -.3.3850 |
| PG0648 | iron compound ABC transporter, periplasmic iron compound-binding protein, putative | Transport and binding proteins | -.3.3537 |
| PG1745 | phosphoribulokinase family protein | Unknown function | -.3.2871 |
| PG1899 | TonB-dependent receptor, putative | Transport and binding proteins | -.3.2836 |
| PG1497 | DNA-binding protein, histone-like family | DNA metabolism | -.3.2693 |
| PG0045 | heat shock protein HtpG | Protein fate | -.3.2641 |
| PG1898 | transporter, putative | Transport and binding proteins | -.3.2263 |
| PG0280 | ABC transporter, permease protein, putative | Transport and binding proteins | -.3.1834 |
| PG0520 | chaperonin, 60 kDa | Protein fate | -.3.1512 |
| PG0283 | efflux transporter, MFP component, RND family | Transport and binding proteins | -3.1367 |
| PG1432 | sensor histidine kinase | Signal transduction | -.3.0989 |
| PG2185 | transporter, putative | Transport and binding proteins | -.3.0958 |
| PG1019 | lipoprotein, putative | Cell envelope | -.3.0536 |
| PG0553 | extracellular protease, putative | Protein fate | -.3.0365 |
| PG1181 | transcriptional regulator, tetR family | Regulatory functions | -.3.0278 |
| PG0919 | dihydroorotase | Purines, pyrimidines, nucleosides, and nucleotides | -.3.0263 |
| PG0194 | ISPg3, transposase | Mobile and extrachromosomal element functions | -.2.9641 |
| PG1896 | S-adenosylmethionine synthase | Central intermediary metabolism | -.2.9333 |
| PG0566 | DNA-binding protein, histone-like family | DNA metabolism | -.2.9035 |
| PG0928 | response regulator | Signal transduction | -2.8670 |
| PG0925 | thymidine kinase | Purines, pyrimidines, nucleosides, and nucleotides | -2.7934 |
| PG0923 | ribosome-binding factor A | Transcription | -.2.7702 |
| PG1485 | conjugative transposon protein TraC | Mobile and extrachromosomal element functions | -2.7396 |
| PG1956 | 4-hydroxybutyrate CoA-transferase | Energy metabolism | -2.7127 |
| PG0010 | ATP-dependent Clp protease, ATP-binding subunit ClpC | Mobile and extrachromosomal element functions | -2.7098 |
| PG2199 | ABC transporter, ATP-binding protein, putative | Transport and binding proteins | -2.6592 |
| PG0173 | transcriptional regulator, putative | Regulatory functions | -2.6590 |
| PG2107 | thiH protein | Biosynthesis of cofactors, prosthetic groups, and carriers | -2.6516 |
| PG2186 | transcriptional regulator, putative | Regulatory functions | -2.6316 |
| PG0540 | AcrB/AcrD/AcrF family protein | Cellular processes | -.2.6254 |
| PG1337 | umuD protein | DNA metabolism | -2.6236 |
| PG0917 | GtrA family protein | Unknown function | -2.6201 |
| PG0181 | immunoreactive 32 kDa antigen PG49 | Unknown function | -.2.6059 |
| PG1660 | RNA polymerase sigma-70 factor, ECF subfamily | Transcription | -.2.5917 |
| PG0158 | competence protein F-related protein | Unknown function | -2.5218 |
| PG1992 | glucose-inhibited division protein A | Unknown function | -2.5193 |
| PG1176 | ABC transporter, ATP-binding protein, putative | Transport and binding proteins | -2.5130 |
| PG1571 | metallo-beta-lactamase superfamily protein | Unknown function | -2.5012 |
| PG2133 | lipoprotein, putative | Cell envelope | -2.4951 |
| PG1063 | transcriptional regulator, putative | Regulatory functions | -2.4688 |
| PG2224 | membrane protein, putative | Cell envelope | -2.4584 |
| PG1478 | conjugative transposon protein TraK | Mobile and extrachromosomal element functions | -.2.4284 |
| PG0279 | NADP-dependent malic enzyme | Energy metabolism | -2.4227 |
| PG1315 | peptidyl-prolyl cis-trans isomerase SlyD, FKBP-type | Protein fate | -2.4145 |
| PG1551 | hmuY protein | Transport and binding proteins | -.2.3936 |
| PG1473 | conjugative transposon protein TraQ | Mobile and extrachromosomal element functions | -.2.3841 |
| PG1038 | ATP-dependent DNA helicase UvrD/PcrA/Rep Family | DNA metabolism | -2.3796 |
| PG0411 | hemagglutinin, putative | Cellular processes | -2.3687 |
| PG1326 | hemagglutinin, putative | Cellular processes | -2.3663 |
| PG0647 | iron compound ABC transporter, permease protein | Transport and binding proteins | -.2.3484 |
| PG1582 | batA protein | Cellular processes | -2.3457 |
| PG1173 | YkgG family protein | Unknown function | -.2.3398 |
| PG0184 | ISPg1, transposase | Mobile and extrachromosomal element functions | -2.3302 |
| PG2032 | primosomal protein N | DNA metabolism | -2.3163 |
| PG1665 | ABC transporter, permease protein, putative | Transport and binding proteins | -2.2999 |
| PG0549 | ISPg1, transposase | Mobile and extrachromosomal element functions | -2.2567 |
| PG1821 | cytochrome c nitrite reductase, small subunit  NrfH | Central intermediary metabolism | -2.2543 |
| PG1259 | anaerobic ribonucleoside-triphosphate reductase activating protein | Purines, pyrimidines, nucleosides, and nucleotides | -.2.2489 |
| PG2108 | thiG protein | Biosynthesis of cofactors, prosthetic groups, and carriers | -.2.2457 |
| PG0838 | integrase | Mobile and extrachromosomal element functions | -2.2427 |
| PG0352 | sialidase, putative | Cell envelope | -.2.2102 |
| PG1664 | ABC transporter, permease protein, putative | Transport and binding proteins | -.2.1972 |
| PG0282 | ABC transporter, ATP-binding protein | Transport and binding proteins | -2.1880 |
| PG1106 | UDP-N-acetylmuramoylalanyl-D-glutamyl-2, 6-diaminopimelate--D-alanyl-D-alanyl ligase | Cell envelope | -2.1859 |
| PG0293 | secretion activator protein, putative | Protein fate | 2.1807 |
| PG2200 | TPR domain protein | Unknown function | -2.1754 |
| PG0192 | cationic outer membrane protein OmpH | Cell envelope | -2.1517 |
| PG0924 | 5'-nucleotidase, lipoprotein e(P4) family | Biosynthesis of cofactors, prosthetic groups, and carriers | -2.1290 |
| PG0932 | DNA polymerase III, delta prime subunit,  putative | DNA metabolism | -2.1168 |
| PG1474 | conjugative transposon protein TraO | Mobile and extrachromosomal element functions | -2.1153 |
| PG2038 | N-acetylmuramoyl-L-alanine amidase, putative | Cell envelope | -.2.1147 |
| PG0750 | glycosyl transferase, group 2 family protein | Cell envelope | -2.1080 |
| PG0864 | site-specific recombinase, resolvase family | DNA metabolism | -2.1072 |
| PG1017 | pyruvate phosphate dikinase | Energy metabolism | -2.1055 |
| PG0281 | ABC transporter, permease protein, putative | Transport and binding proteins | -2.0953 |
| PG1950 | membrane protein, putative | Cell envelope | -2.0758 |
| PG0349 | hydrolase, haloacid dehalogenase-like family | Unknown function | -2.0706 |
| PG1036 | excinuclease ABC, A subunit | DNA metabolism | -2.0685 |
| PG0193 | cationic outer membrane protein OmpH | Cell envelope | -2.0668 |
| PG2215 | mannose-1-phosphate guanylyltransferase | Cell envelope | -2.0509 |
| PG0857 | transcriptional regulator, putative | Regulatory functions | -2.0421 |
| PG0088 | peptidase, M16 family | Protein fate | -2.0295 |
| PG1663 | ABC transporter, ATP-binding protein | Transport and binding proteins | -2.0256 |
| PG1320 | ISPg1, transposase, internal deletion | Disrupted reading frame | -2.0256 |
| PG1433 | hydrolase | Unknown function | -2.0212 |
| PG2035 | tRNA (guanine-N1)-methyltransferase | Protein synthesis | -2.0104 |
| PG1897 | thiamin pyrophosphokinase catalytic domain  protein | Biosynthesis of cofactors, prosthetic groups, and carriers | -2.0104 |

^a^: Locus number, putative identification and cellular role are according to TIGR genome database.

^b^: Average fold difference indicates the expression of the gene by antimicrobial peptide Nal-P-113 addition versus no antimicrobial peptide Nal-P-113 addition.
